# Efficient Demulsification Performance of Emulsified Condensate Oil by Hyperbranched Low-Temperature Demulsifiers

**DOI:** 10.3390/molecules28227524

**Published:** 2023-11-10

**Authors:** Shaohui Jiang, Qingsong Li, Qiang Ma, Botao Xu, Tao Zou

**Affiliations:** 1 State Key Laboratory of Heavy Oil Processing, China University of Petroleum East China, Qingdao 266580, China; b16030104@s.upc.edu.cn; 2 Petroleum Engineering Technology Research Institute, Shengli Oil Field Branch, Sinopec, Dongying 257000, China; 3 CNPC Chuanqing Drilling Engineering Company Limited, Chengdu 610051, China; maqsx_sc@cnpc.com.cn; 4 China Oilfield Services Limited, Tianjin 300450, China; xubt@cosl.com.cn; 5 Huabei Oilfield Company, China National Petroleum Corporation, Renqiu 062552, China; cy3_zout@petrochina.com.cn

**Keywords:** low-temperature demulsification, polyether demulsifier, quaternary ammonium salt, site operation

## Abstract

Focusing on the problem of poor demulsification performance of light crude oil emulsions in low-permeability oilfields at low temperatures, the composition of the emulsion samples, clay particle size distribution, and the viscosity–temperature relationship curve of samples were analyzed. Based on the results of emulsion composition analysis and characteristics, the bottle test method was used to analyze the demulsifying effect of different commercial types of demulsifiers, revealing the demulsification mechanism. The field tests confirm the demulsification capabilities of Polyoxyethylene polyoxypropylene quaternized polyoxyolefins surfactants (PR demulsifiers). The results reveal that PR demulsifiers combine the features of decreasing the interfacial tension between oil and water and adsorbing SiO_2_, allowing for quick demulsification and flocculation at low temperatures. This research serves as a theoretical and practical foundation for the study and advancement of low-temperature demulsification technology in oilfields.

## 1. Introduction

Low-permeability reservoirs have extensively spread and have substantial resource potential [1,2]. Low-permeability reservoirs are usually developed by chemical displacement and seepage [3]. Despite the low viscosity of light crude oil and the low-colloidal asphaltene dosage, a significant number of polymer additives in the oilfield production process has increased emulsion stability, increasing the difficulty of demulsification [4,5]. Heat treatment, chemical demulsification, physical demulsification, and biological demulsification are frequent methods, with chemical demulsification technology being the most extensively utilized in oilfields. Demulsifiers reduce the surface tension of the interfacial film by changing the type of emulsion to affect the interfacial characteristics, destabilizing and breaking the crude oil emulsion [6,7,8]. The irreversible aggregation of the droplet into one or more large area is a vital phase in the demulsification process. The demulsification process comprises flocculation, coagulation, and settlement separation, all of which are carried out concurrently [9,10].

Crude oil demulsifiers are frequently surfactants, and their interfacial activity is greater than that of crude oil film-forming chemicals, which can adsorb or partially replace emulsifiers in the interfacial membrane. The newly produced interfacial coating has outstanding hydrophilic characteristics and lower interfacial strength, which promotes emulsion breaking [11,12,13]. Conventional thermochemical dehydrating crude oil demulsifiers are now being researched and used widely. The crude oil dehydrating method of end-point dosing, pipeline emulsion breaking, and centralized settling is often used in low-permeability oilfields, and the emulsion breakers selected at the site have good emulsion breaking effect at higher temperatures (the range of temperature is from 60 °C to 70 °C) [14]. Currently, demulsifiers with green environmental protection, high-efficiency dehydrating, low-temperature demulsification, and low prices have become increasingly popular in order to maximize resource utilization and reduce environmental damage [15]. Furthermore, low-temperature demulsifiers allow the collection system to run without heating in the summer and with heating to room temperature in the winter, lowering energy consumption and CO_2_ emissions while providing economic benefits [16]. However, the widely used demulsifier’s low-temperature emulsion-breaking performance is inadequate. The emulsion layer of the settling tank after breaking the emulsion is excessively thick, and the water content in the overflow port is likewise high, affecting the gathering system’s regular operation. Because crude oil emulsions are derived from various production blocks, the mechanism of emulsification and stabilization of extracted crude oil, the relationship between different emulsions and the effect of emulsion breaking, and the mechanism of action of low-temperature emulsion breakers are all insufficiently defined [17,18]. Common low-temperature demulsifiers include ester copolymers [19], derivatives of highly polar organic ammonia [20], hydrophobically linked trimers [21], silicone-containing demulsifiers [22], polyethylene polyamine block polyethers [23], and phenol-amino aldehyde resin polyether demulsifiers [24]. Low-temperature demulsifiers achieve rapid demulsification of emulsions in low-temperature environments by decreasing the viscosity of the emulsion, increasing the dispersion rate of the demulsifier in the emulsion, and limiting the influence of waxes in low-temperature environments.

The influence of temperature on the emulsion viscosity and density differential between oil and water is the fundamental cause of the difficulty in breaking crude oil emulsion at low temperatures [25]. The viscosity of crude oil is higher at low temperatures than at high temperatures, and the diffusion and adsorption process of demulsifiers in crude oil is hampered, resulting in a decreased migration rate of emulsion breakers. Furthermore, the density difference between oil and water becomes lower at low temperatures, which influences the settlement and separation of liquid beads due to the density difference [26,27]. For emulsion mixtures containing solid-phase particles, the particles also affect the demulsification effect. Since solid particles are easily adsorbed by negatively charged crude oil, the particles are suspended in the crude oil, which affects the settling and separation of oil and water after demulsification [28]. Therefore, for light crude oil emulsions containing a large number of particles, there are mainly mechanical and chemical methods to realize emulsion solid–liquid phase separation [29]. The chemical method is through the demulsifier and inorganic or organic macromolecular compounds compound, at the same time prompting the destabilization of suspended particles and oil–water interfacial membrane destabilization, solid-phase particle flocculation and settlement, oil droplets and droplet agglomeration separation, and ultimately promoting the separation of the water phase, the oil phase, and the solid phase [30]. After the dissociation of flocculants in emulsion, the counter-ion with the opposite charge of oil droplets diffuses to the surface of oil droplets, compresses the double electric layer, and reduces the electric repulsion between oil droplets, so that the dispersed phase can be aggregated due to van der Waals’ gravity, and reduces the stability of emulsification [31].

New multifunctional oilfield demulsifiers have been investigated to solve the problem of increasing water content in oilfield recovery fluids [32]. The multifunctionality of oilfield demulsifiers is usually achieved by compounding multiple single reagents. Bhupati R. Bhattacharyya has proposed the synthesis of copolymer demulsifiers by emulsion polymerization using unsaturated acids and their corresponding derivatives as raw materials, containing many hydrophilic and lipophilic groups, where other functional groups can be introduced into the side chain to achieve the multifunctionality of the demulsifiers [33]. Zhai prepared a new type of demulsifier with a synergistic effect of demulsification and adsorption of oil droplets by silica gel-loaded polyether polysiloxane quaternary ammonium salt, which is conducive to the improvement of the sedimentation and separation rate in the late stage of demulsification [22]. It can also introduce other functional groups into the side chain to realize the multifunctionality of the demulsifier.

In this research, a collection of fluid components and properties was studied to investigate demulsifying mechanisms based on the usual low-permeability reservoir circumstances of a particle. The simulated emulsion was subjected to demulsifier optimization at low temperatures (25 °C, compared to conventional high-temperature dehydration) to generate a low-temperature demulsifier formulation suitable for this low-permeability reservoir. The optimization of a low-temperature demulsifier provides a theoretical and practical foundation for emulsion breaking and dehydrating low-permeability oilfield extraction fluids.

## 2. Results and Discussion

### 2.1. Emulsified Condensate Characterization

The primary objective of this work is to optimize chemical demulsifiers that can efficiently break emulsification at low temperatures (20–30 °C), but treat large volumes of liquids in the field, making low-temperature emulsification difficult. The emulsion components are shown in Table 1. The field sample emulsion comprised 0.507 water, 0.384 straight-chain hydrocarbons and cycloalkanes, 0.056 clay content, 0.014 sodium and 0.019 calcium salts, and 0.02 surface-activating agent, such as dodecyl-dimethyl propylammonium chloride. Dodecyldimethylpropylammonium chloride in the emulsion component is a category of substances with emulsifying properties formed by the reaction of oilfield admixtures during extraction and transportation. The extracted fluid is sheared at a high speed to form an emulsion, and the surface activator and the clay material enhance the interfacial tension, resulting in increased difficulty in breaking the emulsion at room temperature.

**Figure 1:**
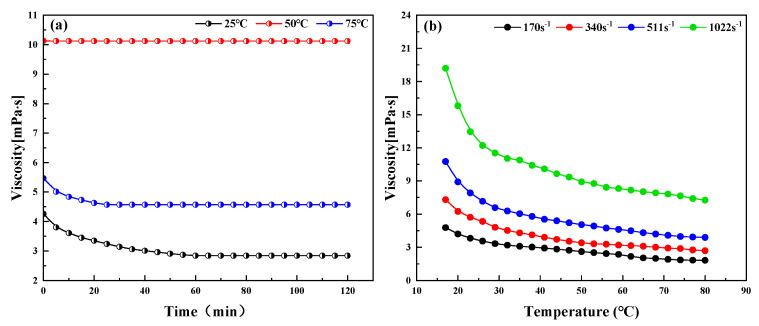
Viscosity–temperature curves of crude oil sample from low–permeability oilfield. (**a**) Effect of temperature on viscosity; (**b**) effect of shear rate on the viscosity–temperature profile of emulsions.

The impact of different temperatures on viscosity and the impact of shear rate on the viscosity–temperature curve were tested by a DV-II viscometer to examine the anomalies of the emulsions. Figure 1a shows the trend of emulsion viscosity with time at 25 °C, 50 °C, and 75 °C. The emulsion viscosity does not vary with time at 25 °C, indicating that the emulsion is stable. At 50 °C or 75 °C, the emulsion viscosity tends to decrease and then stabilize as the test time increases, and the decrease in viscosity is less, indicating that the emulsion is partially demulsified in the 50 °C or 75 °C temperature environment. Figure 1b shows the trend of viscosity with temperature under different shear rate conditions. The viscosity of the emulsion under different shear rate conditions decreases with the increase in temperature, and the viscosity decreases greatly in the interval of 17~30 °C. Figure 1 shows the low sensitivity of the emulsion to temperature at low temperatures, with a more pronounced viscosity change only at 50 °C and above. The increase in shear rate decreases the apparent viscosity of the emulsion, but due to the absence of gums and asphaltenes, the anomaly point is not obvious, and there is no sudden viscosity change point. By examining the relationship between viscosity, temperature, and shear rate, it was found that elevating the temperature could not separate the muddy components in the emulsion. Table 1 shows that the emulsion components are free of colloidal asphaltenes, which also indicates that the emulsions do not have the conditions for the presence of a return to the normal point.

**Figure 2:**
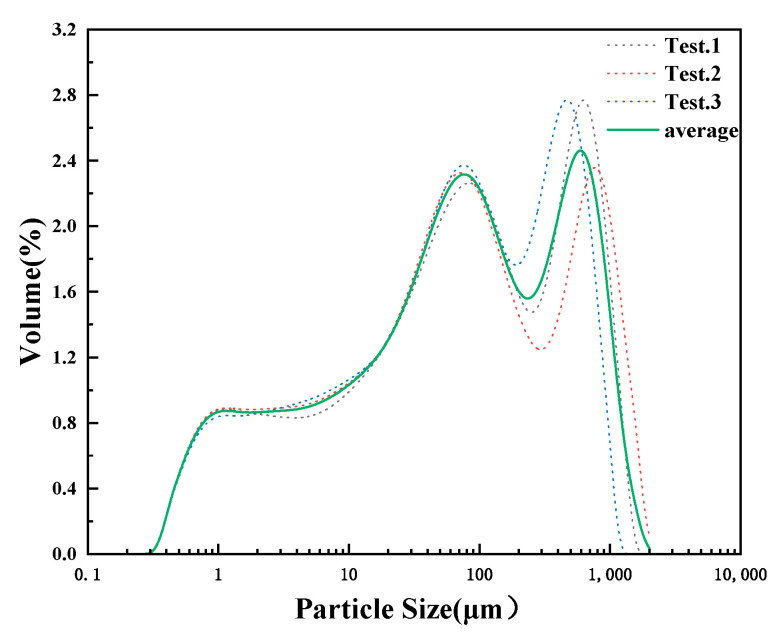
Particle size distribution area of the solid phase.

Since the surface layer of tiny particles in emulsions is charged and affects the boundary surface energy of the emulsion, thus increasing the difficulty of breaking the emulsion, in this paper, the solid-phase materials in emulsions were tested for particle size analysis. Figure 2 shows the particle size distribution of the solid phase; more than 60% of the particles in the emulsion are 1–100 μm in diameter, and the particle size is small. The difficulty in demulsification containing clay micro-particle emulsion is attributed to the adsorption of the particles on the surface of the emulsion which enhances the interfacial strength, while the surface charge of the clay micro-particle facilitates the stabilization of the emulsion. The smaller the particle size of clay particles, the easier it is to be adsorbed on the surface of oil–water droplets, and the more conducive to emulsion stabilization. Therefore, it is necessary to investigate not only the dehydrating rate of the emulsion at room temperature by the emulsion breaker but also the oil removal rate.

### 2.2. Effect of Different Surfactant Demulsifiers on the Demulsification of Emulsified Condensate Oil

**Figure 3:**
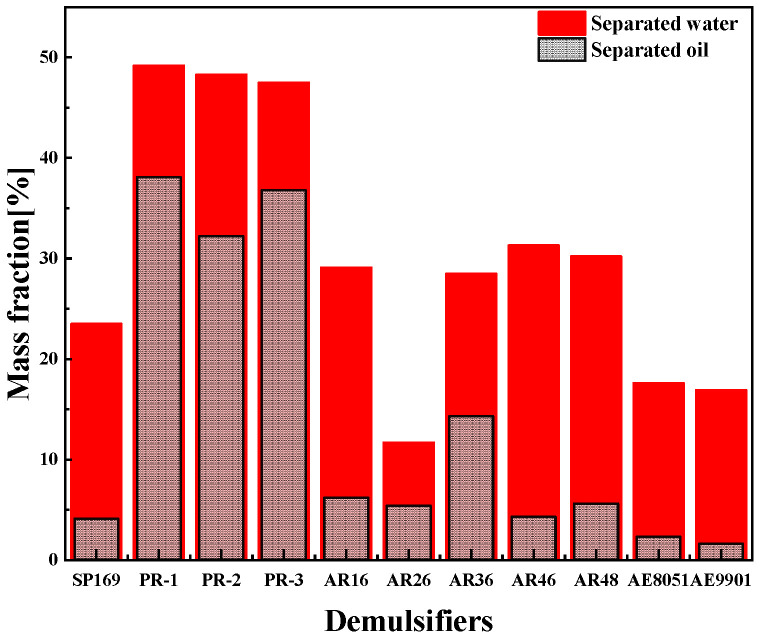
Effect of different demulsifiers on emulsions.

**Figure 4:**
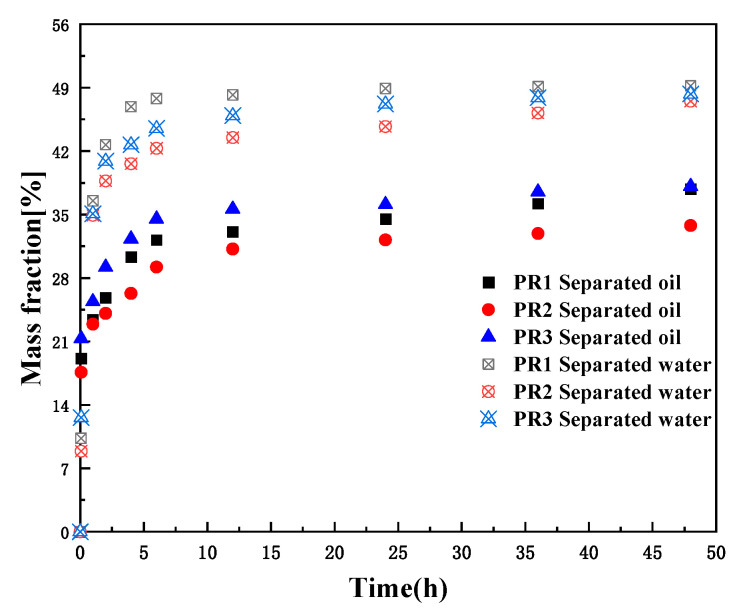
Effect of different surfactant demulsifiers (PR1, PR2, and PR3) on demulsification of emulsions over time. MS stands for oil or water separation.

**Figure 5:**
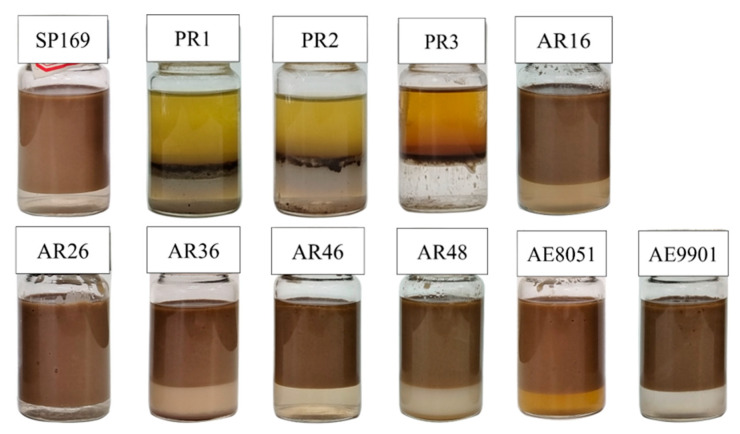
Selection of photographs showing the quality of the separated water after demulsification using, from left to right, SP, PR, AR, and AE.

As shown in Figure 3, different surfactants have different oil and water separation efficiencies, and demulsifiers such as SP, PR, AE, and AR were selected to measure the demulsification experiments at a constant temperature of 25 °C. The experimental results showed that the demulsifier of SP169, AE, and AR had poor demulsification and oil removal performance, and the PR demulsifier had excellent demulsification de-oiling and dehydrating performance. The demulsifier of SP and AR has better performance in dehydrating compared to de-oiling, and the dehydrating, and de-oiling performance of the AE series of demulsifiers are all poor. The PR series demulsifiers could separate more than 60% of the oil at room temperature within the initial 1h and separated all the oil and water within 1 day (Figure 4). The outstanding demulsification performance of the PR series demulsifier is attributed to the introduction of flocculating ionic chain segments on the polyoxyethylene polyoxypropylene block. The cation in surfactants has the capability of destabilizing interfacial charges and flocculating solid-phase particles in emulsions, which can effectively diminish the interfacial strength of the emulsion, accelerate the emulsion breaking efficiency and solid-phase particle flocculation, and significantly enhance the discarding oil efficiently of emulsions. Figure 5 shows that SP169, AE, and AR demulsifiers all have dehydrating performance, however, the upper layer of oil and mud particles cannot be separated, making demulsification difficult.

Figure 4 shows the trend of separated oil and water rates after demulsification over time by adding 800 mg/L of PR1, PR2, and PR3 emulsion breakers at 25 °C. After adding the demulsifiers, the emulsion was rapidly demulsified to remove oil, but a large amount of oil was still not detached, and the demulsification effect increases with emulsion breaking time. The separated water rates after 48 h of breaking the emulsion were 49.2%, 47.5%, and 48.3%, and the separated oil rates were 37.8%, 33.8%, and 38.1%, respectively, for PR1, PR2, and PR3. Among them, PR1 and PR3 had a de-oiled and dehydrated effect consistent with the compositions in the emulsions, and both had superior demulsification effects.

### 2.3. Effect of Different Temperature and Demulsifier Concentration on the Demulsification of Emulsified Condensate Oil

**Figure 6:**
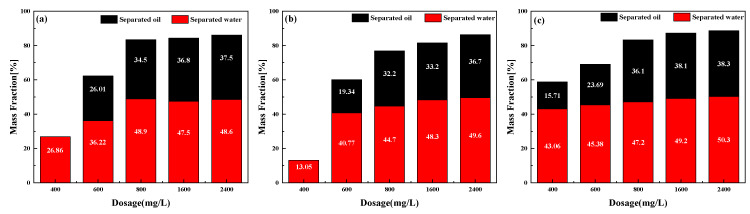
Influence of demulsifier dosage on the emulsion breaking. (**a**) PR1; (**b**) PR2; and (**c**) PR3.

Both temperature and concentration of demulsifiers are significant factors in the demulsification effect. The demulsification effect was investigated by adding different doses of PR1, PR2, and PR3. Figure 6a–c shows the separated oil and separated water rate after 24 h of emulsion breaking at 25 °C with the addition of 800 mg/L demulsifiers, which have an excellent demulsification effect. When the demulsifier dosage was 800 mg/L, the separated water rates were 48.9%, 44.7%, and 47.2%, and the separated oil rates were 34.5%, 32.2%, and 36.1%, respectively, for PR1, PR2, and PR3. Compared to the demulsifier dosage of 800 mg/L, the separated oil rate increased by 8.7%, 13.98%, and 6.09% when the demulsifier dosage was 2400 mg/L. In summary, it is observed that the de-oiling and dehydrating efficiency increased by 6.09% when the dosage was more than 800 mg/L, and the dehydrating effect of PR3 was optimal. Therefore, the demulsifier concentration of PR3 can be adjusted according to economic efficiency.

**Figure 7:**
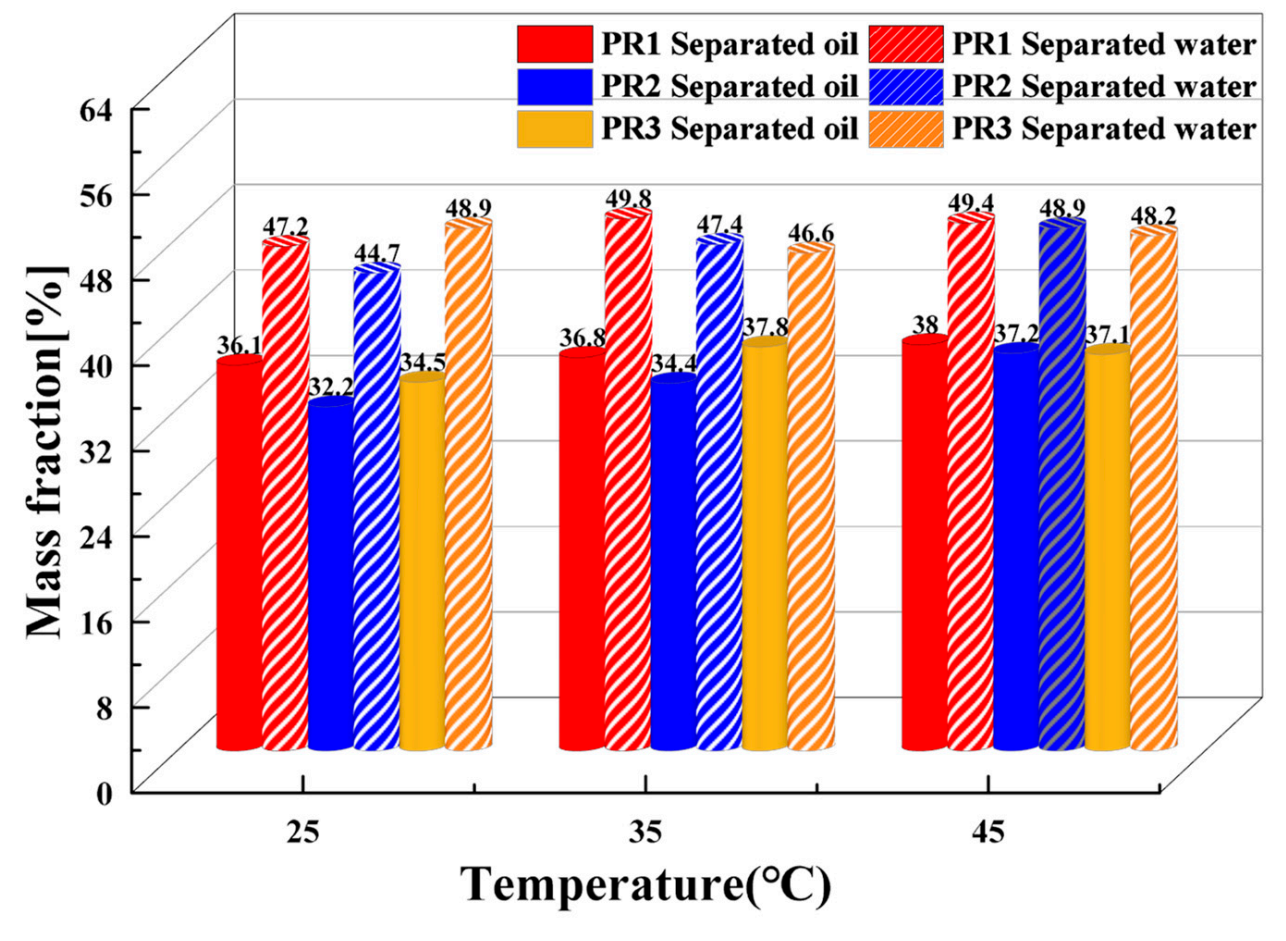
Influence of temperature on the performance of demulsifiers (PR1, PR2, and PR3).

Due to the large treatment capacity of oilfield emulsion, and the low temperature in winter, usually below 0 °C, the heating capacity of field equipment is poor; thus, we examined the effect of temperature on the demulsification effect of PR series demulsifiers. Figure 7 shows the demulsification effect after 24 h at 25 °C, 35 °C, and 45 °C. The concentrations of the demulsifiers were all 800 mg/L. The dehydrating and removal oil rate of the PR demulsifier was enhanced with the increase in the demulsification temperature, among which the PR3 demulsifier had the most optimal demulsification effect.

### 2.4. Demulsifier Performance and Dehydration Mechanisms of the Emulsified Condensate Oil

**Figure 8:**
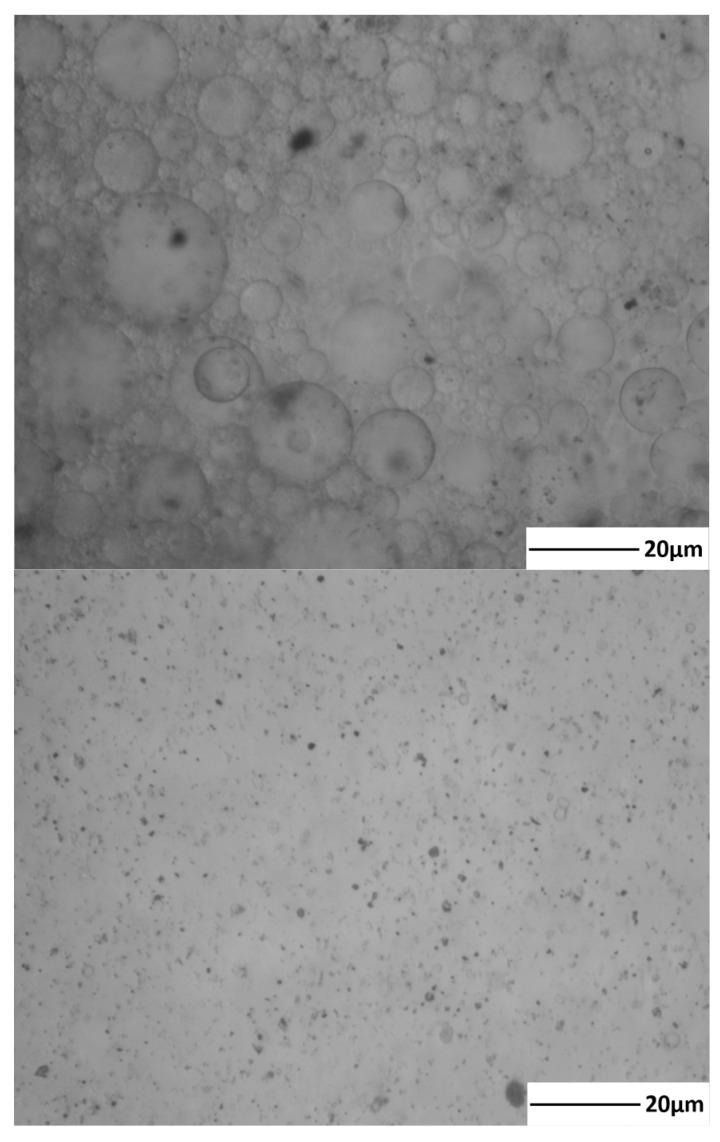
Micrograph of emulsion before and after emulsion breaking.

Owing to the different surfactants used during the production process, the stability of the emulsion is enhanced after high-speed shear rate conditions during transportation, complicating the demulsification. The study shows that the difficulty in the demulsification of an emulsion depends on the strength of the oil–water interfacial film. The stronger the interfacial film and the slower the dispersion speed of the demulsifier, the more difficult it is to demulsify the emulsion [34]. It is well known that due to the negative charge of oil droplets in water, oil droplets repel each other to form a stable emulsion. The compositional analysis (Table 2) and microscopic observation (Figure 8) of the samples revealed that the adsorbed 1–100 μm clay particles at the interface of the oil–water phase enhanced the interfacial tension, which increased the difficulty in emulsification of the emulsions, in addition to the slowing down of the dispersion of the emulsifier in the emulsions at low temperatures, which also reduced the emulsification efficiency of the emulsions.

**Figure 9:**
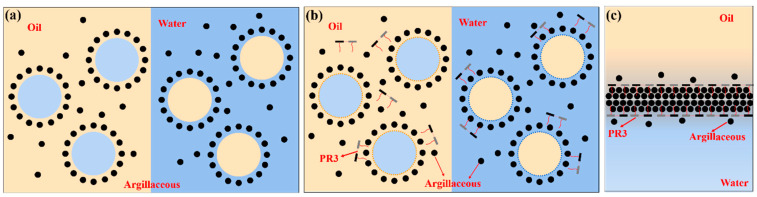
Emulsion breaking mechanism diagram. (**a**) Stable emulsion, (**b**) Demulsification (**c**) Stratification.

The process of oil–water interfacial film from formation to rupture after adding the demulsifier is shown in Figure 9. The cationic chains in the PR demulsifier adsorbed on the oil–water interface promote the rupture of the interfacial film, and the emulsion droplets are more likely to collide, which leads to the destabilization of the emulsion. The mechanism of the demulsifier in reducing the strength of the interfacial membrane mainly includes the following: The interfacial membrane is easier to rupture by reducing the interfacial shear viscosity to release dispersed-phase droplets. The released droplets are more likely to collide with the aggregation and destabilize the emulsion by reducing the interfacial membrane viscoelasticity to achieve the effect of oil–water separation. The solid–liquid phase separation speed was promoted by the flocculation of clay particles [35,36].

Polyether demulsifiers are surface activators with adsorption on the oil–water interface through the topping effect and produce a new oil–water interface [37]. The thickness of the newly formed interfacial membrane is thinner. The strength of the membrane is significantly decreased and emulsification occurs [24]. In addition, it has been shown that SiO_2_ with adsorption function is embedded in the polyether emulsion breaker. The PR demulsifier was modified for quaternary ammonium salts with an SiO_2_ adsorption function on polyether emulsion breakers [38]. PR demulsifiers provide the capability to reduce the viscoelasticity of the interfacial film and the interfacial shear viscosity and flocculate the clay particles. The PR emulsion breaker first displaces and destroys the emulsion membrane, after the completion of emulsion breaking, the oil droplets can be adsorbed by the positive charge quaternary ammonium salt with flocculation function to complete the emulsion breaking and flocculation in the same period.

### 2.5. The Experiment of On-Site Low-Temperature Demulsification

**Figure 10:**
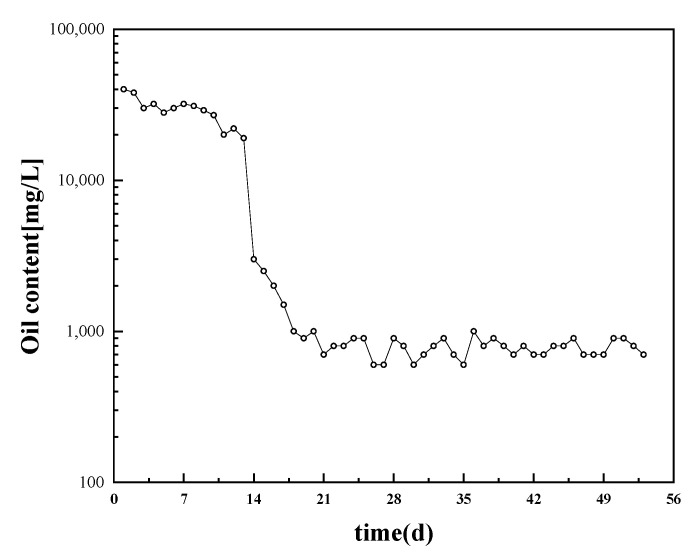
Variation of water content after emulsion breaking and separation.

The output liquid first enters each metering station. All metering stations enter the mixing pumping station, then it is pressurized by mixing pumps and finally enters the three-phase separator of the first station through the external transport from the mixing pumping station to the first station. After entering the three-phase separator, the separated water is designed to contain less than 1000 mg/L of oil. The dosage of crude oil demulsifier is added at the first end as much as possible to provide sufficient time to break the emulsion. In summary, the emulsion-breaking performance experiments show that the PR3 demulsifier is optimal, and the dosing concentration at each metering station is recommended to be 800 mg/L. The variation of water content in the first station was monitored daily during the field test, and the test results are shown in Figure 10. After adding PR3, the oil content of the separated water from the three-phase separator decreased rapidly from 19,000 mg/L to less than 3000 mg/L, and to 1000 mg/L after 5 days.

## 3. Materials and Methods

### 3.1. Materials

**Figure 11:**
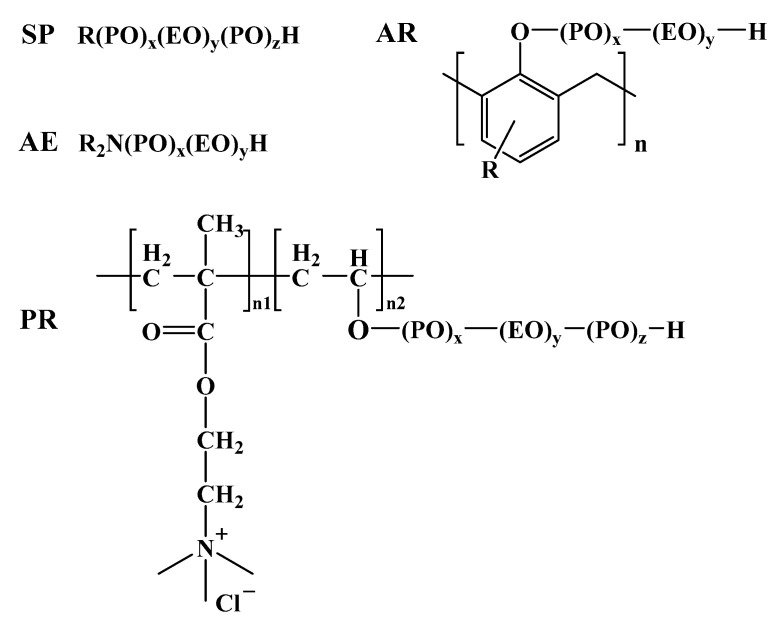
Chemical structure of demulsifies (x, y, z, and n is the degree of polymerization of different demulsifies, R stands for alkyl).

The emulsified condensate oil used in this investigation was sourced from the Dongsheng gas field in China, and its parameters are described in Table 1. (Figure 11) Polyethylene polyamine polyoxyethylene polyoxypropylene ether (AE9901, AE8051), Polyoxypropylene polyoxyethylene propylene glycol ether (SP169), Alkyl phenol formaldehyde resin polyoxypropylene polyoxyethylene ether (AR16, AR26, AR36, AR46, and AR48), and Polyoxyethylene polyoxypropylene quaternized polyoxyolefins (PR-1, PR-2, and PR-3) were graciously provided by the Shengli Chemical Co. Ltd. (Dongying, China), and demulsifiers are from the commercially available series. The above drugs are of industrial purity. Table 1 summarizes the primary functionality, relative solubility number (RSN), and terminology used in this work.

### 3.2. Relative Solubility Number Determination

The RSN of the demulsifier was estimated in this research by titration experiments with deionized water to assess the hydrophilicity and hydrophobicity of demulsifiers. The mixed solution of toluene and dimethoxyethane was prepared with a volume ratio of 2.6%/97.4% (*v/v*). Then, 1.5 g demulsifiers were weighed and added to the mixed solution until a cloudy solution was obtained, which was maintained for 30 s. Finally, titration was initiated until turbidity 26 was attained, and three observations were averaged using the preset parameters [23].

### 3.3. Bottle Tests

The solution was transferred to a glass container (25 mL) by weighting 20 g of emulsion samples and was tested for several types of demulsifiers at a constant temperature (25 °C). A total of 18 mg of demulsifier was introduced to the emulsion by using a micro-injector, followed by mixing the demulsifiers and the emulsion by vibrating it for 5 min to fully combine. The glass vials were returned to the thermostat at 25 °C, and the mass of oil and water, which was separated, was weighed at regular intervals to account for emulsion breakdown and separation.

The performance of the demulsifier is measured in terms of the mass percentage of separated water (MS), wt %, which is defined as

Water/oil separation (m/m%) = m_i_/m_0_ × 100 (1)

where m_i_ is the mass of the water or oil separated and m_0_ is the original mass of water or oil contained.

### 3.4. Measurement of Viscosity–Temperature Curve and Abnormal Point of Dehydrated Crude Oil

The beaker containing about 150 g of sampled emulsion was heated in a thermostatic water bath, after which the viscosimeter was opened and initialized. The system and sample were heated to 80 °C, a constant temperature for 10 min to start the measurement. The test temperature was reduced at a rate of 2~5 °C/min until 25 °C, and the temperature was maintained at a constant temperature for 10 min after each temperature was reached. The shear viscosity of the emulsions was measured at a shear rate in the range of 170–1022 s^−1^. The anomaly point of the emulsion was analyzed by observing the position of the inflection point of the viscosity–temperature curve.

### 3.5. Measurement of Clay Particle Size

The emulsion sample was separated from the oil, water, and solid phases by high-speed centrifugation, and the separated clay solid phase was placed in a 100 °C constant-temperature drying oven for 48 h to obtain the experimental sample. Weighing 100 mg of the sample into the mechanical stirring cell, ultrasonic high-frequency vibration was used to make the agglomerated particles fully dispersed and an electromagnetic circulation pump was used to make the size of the particles dispersed throughout the entire circulatory system in order to obtain a wide distribution of samples to test the accurate repeatability of the results. Clay particle samples were measured using the laser particle sizer (BT-9300H(T), Dandong Bettersize Instruments Co., Ltd. Dandong, Liaoning in case of China) to obtain the distribution of their particle sizes.

### 3.6. Observation of Emulsion Microstructure

A total of 2 mL of sample was dropped in the center position of a clean and dry slide. A coverslip was gently squeezed to avoid air bubbles, and then the focus of the microscope was adjusted to find the target sample. The micro-morphology of the emulsion samples before and after demulsification was observed by the M230-type metallographic microscope (Shenzhen AOSVI Optical Instrument Co., LTD. Shenzhen, Guangdong in case of China).

## 4. Conclusions

The reasons for the emulsion demulsification difficulty at room temperature were analyzed by researching the composition of the emulsion samples, clay particle size distribution, and the viscosity–temperature relationship curve of samples. The demulsification effect of several types of surfactants on crude oil emulsions at low temperatures was investigated by the bottle-testing method, and the demulsification mechanism was analyzed in combination with microscopic experiments. The experimental results revealed that the emulsion samples did not contain colloidal asphaltenes, thus there was no abnormal point; however, there were a large number of muddy particles ranging from 1 to 100 μm, which increased the difficulty of low-temperature emulsion breaking. PR demulsifiers combine the features of decreasing the interfacial tension between oil and water and adsorbing SiO_2_, allowing for quick demulsification and flocculation at low temperatures. With the PR3 emulsion breaker used for field experiments, the oil content of the separated water can reach 3000 mg/L in 1 day, and after 5 days of continuous emulsion breaking, it can reduce to 1000 mg/mL.

## Figures and Tables

**Table 1:** Emulsion components and properties.

	Parameter	Mass Ratio/%
Mass density/Kg‧m^−3^ at 20 °C	891	
Viscosity/mPa‧s at 20 °C	4.6	
Mass fraction of water		0.507
Mass fraction of alkane	C_5_–C_21_	0.308
	Naphthenic hydrocarbon	0.076
Mass fraction of salt	Sodium chloride	0.014
	Calcium chloride	0.019
Mass fraction of argillaceous		0.056
Surfactants		0.02

**Table 2:** Demulsifiers used in this work, their series, and relative solubility number.

NO.	Demulsifier Series	Name	RSN	R	x,y,z
1	SP	SP169	10.3	18	(200–800, 400–1000, 600–1200)
2	PR	PR-1	13.3	-	(300–500, 200–500, 300–500)
3		PR-2	12.6	-	(500–800, 500–700, 500–700)
4		PR-3	12.8	-	(500–800, 700–1000, 700–1000)
5	AR	AR16	12.4	6	(300–1000, 200–400, 0)
6		AR26	12.8	6	(300–1000, 300–500, 0)
7		AR36	13.6	6	(300–1000, 400–600, 0)
8		AR46	15	6	(300–1000, 500–700, 0)
9		AR48	15.2	6	(300–1000, 600–800, 0)
10	AE	AE9901	13	12	(300–600, 200–500, 0)
11		AE8051	17	12	(500–1000, 500–800, 0)

## Data Availability

No new data were created or analyzed in this study. Data sharing is not applicable to this article.
